# Supplementary material for: Radiometer calibration using machine learning
Source: Sci Rep. 2025 Oct 2;15:34335. doi: 10.1038/s41598-025-16732-9 (PMC12491404; doi:10.1038/s41598-025-16732-9)
Supplement: Supplementary file 1 — Supplementary Information. [file 41598_2025_16732_MOESM1_ESM.pdf]

1 Supplementary Information A: Assumptions In Noise Wave Parameter Methods

When calibrating a receiver in the Noise Wave Parameter framework, the reflection coefficients of the calibration source ($\Gamma_{\rm cal}$) and the receiver itself ($\Gamma_{\rm rec}$) are measured, along with the power spectral densities (PSDs) of the calibration source ($P_{\rm cal}$), the internal reference load (P_L), and the internal noise source ($P_{\rm NS}$) [1, 2]. These measurements allow for a preliminary, uncalibrated temperature calculation, commonly denoted $T^*_{\rm cal}$, to be computed via Dicke-switching using:

$$T^*_{\rm cal} \;=\; T_{\rm NS}\left(\frac{P_{\rm cal} \;-\; P_L}{P_{\rm NS} \;-\; P_L}\right) \;+\; T_L, \tag{1}$$

where T_L is an assumed value for the noise temperature of the internal reference load, and $T_{\rm NS}$ is the assumed excess noise temperature of the internal noise source. This step removes system gain variations arising from cables, filters, amplifiers, and the analogue-to-digital converter [1], under the assumption that...

Each PSD measurement may then be written in the form:

$$\begin{aligned} P_{\rm cal} \;=\; g_{\rm sys}\Big[& T_{\rm cal}\big(1-|\Gamma_{\rm cal}|^2\big)\left|\frac{\sqrt{1-|\Gamma_{\rm rec}|^2}}{1-\Gamma_{\rm cal}\Gamma_{\rm rec}}\right|^2 \;+\; T_{\rm unc}\,|\Gamma_{\rm cal}|^2\left|\frac{\sqrt{1-|\Gamma_{\rm rec}|^2}}{1-\Gamma_{\rm cal}\Gamma_{\rm rec}}\right|^2 \\ & +\; T_{\rm cos}\,\Re\left[\frac{\Gamma_{\rm cal}}{1-\Gamma_{\rm cal}\Gamma_{\rm rec}}\right]\sqrt{1-|\Gamma_{\rm rec}|^2} \;+\; T_{\rm sin}\,\Im\left[\frac{\Gamma_{\rm cal}}{1-\Gamma_{\rm cal}\Gamma_{\rm rec}}\right]\sqrt{1-|\Gamma_{\rm rec}|^2} \;+\; T_0\Big], \end{aligned} \tag{2}$$

where $g_{\rm sys}$ is the system gain referenced to the receiver input, and $T_{\rm cal}$ denotes the calibrated input temperature. The terms $T_{\rm unc}$, $T_{\rm cos}$, and $T_{\rm sin}$ are the "noise wave parameters" introduced by Meys [3], which have been employed in global 21-cm experiments [1, 2, 4]. In these methods, the internal load and noise sources are assumed to have negligible reflection coefficients (often approximated as zero when $|\Gamma| < 0.005$). This is illustrated by:

$$P_L = g^*_{\rm sys}\Big[T_L\big(1-|\Gamma_{\rm rec}|^2\big) + T^*_0\Big], \tag{3}$$

$$P_{\rm NS} = g^*_{\rm sys}\Big[\big(T_L + T_{\rm NS}\big)\big(1-|\Gamma_{\rm rec}|^2\big) + T^*_0\Big]. \tag{4}$$

When the internal references are located on a different reference plane from that of the receiver input, they introduce offsets $g^*_{\rm sys}$ and T^*_0 distinct from the values in the main signal path [1]. EDGES-style approaches account for these using extra scale and offset parameters [1, 2], which can also absorb uncertainties in T_L and $T_{\rm NS}$.

Both the Noise Wave and Noise Parameter methods use two sets of sources: one for Dicke-switching and another for calibration parameter estimation (Noise Wave Parameter or Noise Parameter). The sources used for Dicke-switching must be matched, whereas the sources used for calibration parameter estimation do not need to be matched.

Finally, combining the above expressions produces a linear relationship between the *uncalibrated* temperature and the final, *calibrated* temperature. The reader should note that throughout such treatments, several assumptions are made: (i) low-reflection internal references, (ii) negligible mismatch between the internal references, and (iii) parametric modelling of noise waves via polynomials in frequency ($T_{\mathrm{unc}}, T_{\cos}, \ldots$).

In experiments where the required precision presses well beyond the validity regime of these approximations (as is often the case for sky-averaged 21-cm experiments), systematic errors may be introduced. By contrast, our machine learning approach predicts the necessary parameters (specifically, the Noise Parameters) without *a priori* assumptions of perfect impedance matching or negligible reflections in the internal references. Consequently, our framework avoids introducing potential modelling biases inherent in the traditional scheme, providing a more comprehensive solution for high-precision sky-averaged 21-cm radiometry.

2 Supplementary Information B: Training on a Temporally Unstable System

To demonstrate how the method operates on an unstable system, we generated a simulated dataset designed to mimic a temporally unstable system, reflecting the complexities encountered in real-world observational environments. Similar to the process described in Section **??** for generating simulated data, we simulated a realistic model of the receiver chain. However, in this instance, we extended the simulation to cover multiple observations, accumulating a total of 1000 observations, each lasting 30 seconds, for every calibration source. This dataset allows us to test our calibration framework in the temporal domain, predicting three-dimensional Noise Parameter *surfaces* (parameter value as a function of frequency and time), rather than just two-dimensional frequency-dependent curves.

A key distinction in this simulation was the introduction of temporal variability into one of the system's Noise Parameters. Specifically, the Noise Parameter T_{min} was varied sinusoidally by a fixed offset over the course of the observations, fluctuating by $\pm 5\%$ around its nominal value. This variation was implemented to simulate the gradual drifts and changes that can occur in the electronic components of a receiver due to environmental factors or ageing, representing a significant challenge for traditional calibration techniques. It is important to note that many conventional calibration methods rely on the assumption that receiver Noise Parameters remain stable over the observation period, with the exception of the overall gain g. Such temporal instabilities, if not properly accounted for, can introduce systematic errors that corrupt the final scientific measurements.

We trained the neural network using this temporally dynamic dataset. The network successfully learned to characterise the unstable system, demonstrating its ability to adapt to and model time-varying instrumental effects by predicting the full three-dimensional Noise Parameter surfaces. By processing the sequence of observations, the model effectively captured the underlying sinusoidal variation in T_{min} within its predicted surface, whilst simultaneously determining the surfaces for the other stable Noise Parameters and the gain. It is worth noting here that there is some degeneracy

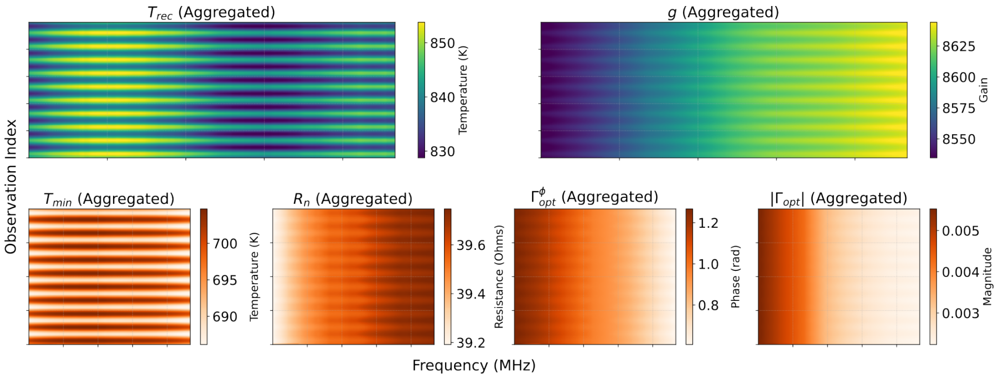

Fig. S1 Heatmaps illustrating the temporal evolution of the inferred receiver Noise Parameters over the simulated multi-night observation period. Each panel corresponds to one of the four Noise Parameters ($T_{\rm min}$, R_N, $|\Gamma_{\rm opt}|$, $\Gamma^{\phi}_{\rm opt}$) and the gain g. The y-axis represents observation order (ascending from first to last), and the x-axis represents frequency. Colour intensity indicates the inferred parameter value. The sinusoidal variation deliberately introduced into $T_{\rm min}$ (bottom-left panel) is clearly visible and accurately recovered by the neural network, demonstrating the model's ability to characterise a temporally unstable system. Note that each heatmap contains aggregated data from all calibrators used in the simulation.

between $T_{\rm min}$ and the other noise parameters, causing a light ripple to appear in the time axis of the other heatmaps. This does not affect the overall calibration result.

Figure S1 illustrates the network's performance in tracking the evolving Noise Parameters over the simulated observation period. The heatmaps display the inferred values for each Noise Parameter as a function of time. Notably, the heatmap corresponding to $T_{\rm min}$ clearly shows the sinusoidal oscillation in the time axis introduced into the simulation.

This result signifies an important extension of radiometer calibration into the temporal domain, potentially for the first time. Our machine learning framework moves beyond static, two-dimensional system characterisation (parameter vs. frequency) to predict three-dimensional Noise Parameter surfaces (parameter vs. frequency and time), learning how the physical properties of the instrument evolve. This capability to model temporal variations is particularly valuable as radio astronomy pushes towards deploying instruments in increasingly remote and challenging environments, both on Earth and in space (e.g., lunar missions like LuSEE-Night [5] or potential orbital concepts such as CosmoCube [6]).

References

[1] Monsalve, R.A., Rogers, A.E., Bowman, J.D., Mozdzen, T.J.: Calibration of the edges high-band receiver to observe the global 21 cm signature from the epoch of reionization. The Astrophysical Journal **835**(1), 49 (2017)

[2] Bowman, J.D., Rogers, A.E., Monsalve, R.A., Mozdzen, T.J., Mahesh, N.: An absorption profile centred at 78 megahertz in the sky-averaged spectrum. Nature **555**(7694), 67–70 (2018)

[3] Meys, R.: A wave approach to the noise properties of linear microwave devices. IEEE Transactions on Microwave Theory and Techniques **26**(1), 34–37 (1978)

[4] Rogers, A.E., Bowman, J.D.: Absolute calibration of a wideband antenna and spectrometer for accurate sky noise temperature measurements. Radio Science **47**(06), 1–9 (2012)

[5] Bale, S.D., Bassett, N., Burns, J.O., Jones, J.D., Goetz, K., Hellum-Bye, C., Hermann, S., Hibbard, J., Maksimovic, M., McLean, R., et al.: Lusee'night': The lunar surface electromagnetics experiment. arXiv preprint arXiv:2301.10345 (2023)

[6] Artuc, K., Acedo, E.d.L.: The spectrometer development of cosmocube, lunar orbiting satellite to detect 21-cm hydrogen signal from cosmic dark ages. arXiv preprint arXiv:2406.10096 (2024)
